# Beyond genuine collaboration: the rise of strategic co-authorship in contemporary academic publishing

**DOI:** 10.1186/s41073-026-00197-z

**Published:** 2026-04-28

**Authors:** Rui Marcelino

**Affiliations:** 1 University of Maia, Maia, Portugal; 2 Research Center in Sports Sciences, Health Sciences and Human Development, CIDESD, Elite Research Community, Maia, Portugal; 3 FPF Academy, Portuguese Football Federation, Oeiras, Portugal

**Keywords:** Authorship ethics, Research integrity, Honorary authorship, Publication pressure, Research assessment

## Abstract

The near-disappearance of single-author publications in scientific literature represents one of the most dramatic shifts in academic publishing over the past two decades. While this trend is often attributed to increased scientific collaboration and research complexity, substantial evidence suggests that systemic publication pressures and metric-based evaluation systems have created incentives for "strategic co-authorship"—practices including honorary authorship, gift authorship, and publication cartels that violate established authorship criteria. This article synthesizes empirical evidence documenting the decline of single-author publications, the prevalence of authorship misconduct, and the systemic drivers underlying these practices. Drawing on bibliometric analyses, prevalence surveys, and studies of academic culture, evidence-based synthesis indicates that addressing authorship requires fundamental reforms to institutional assessment systems, enhanced editorial vigilance, and cultural change in how the academic community values research contributions. The integrity of the scientific record depends on honest attribution of intellectual work, yet current incentive structures systematically undermine this foundational principle.

## Introduction

A cursory examination of contemporary scientific literature reveals a striking phenomenon: single-author publications have become increasingly rare across several disciplines. This dramatic shift in authorship patterns raises fundamental questions about the drivers of scientific collaboration and the integrity of authorship attribution. While multi-authorship often reflects genuine advances in collaborative science, interdisciplinary integration, and research complexity, substantial evidence suggests less benign factors significantly contribute to what has been termed "authorship inflation".

The empirical record is more nuanced than a simple narrative of ever-increasing individual productivity. Large-scale analyses show a systematic decline in single-author publications and substantial growth in co-authorship, but when publication output is adjusted fractionally for the number of co-authors, individual researchers’ publication rates have remained broadly stable over the past century and have even declined in several disciplines since the 1980s [9]. Complementary evidence indicates that current growth in article volume largely reflects increased ‘self-fractionalisation’ of researcher effort across multiple collaborative outputs: authors contribute to more papers per year via collaboration, while the average number of full publications per author has decreased [13]. These findings suggest that genuine collaboration and division of labour account for a considerable share of authorship inflation, but do not preclude the concurrent rise of strategic and unethical authorship practices that are the focus of this commentary. This trend coincides with substantial growth in authorship counts: analyses of major medical journals from 1960 to 2010 document more than three-fold increases in author numbers within specific study types, even after adjusting for study population sizes [44]. Perhaps most tellingly, this authorship inflation occurred *independently* of study complexity, persisting across diverse research designs including cost-effectiveness analyses and observational studies where computational advances might theoretically reduce rather than increase collaborative needs [44].

This commentary synthesizes empirical evidence testing the hypothesis that publication pressures and metric-based evaluation systems have incentivized strategic co-authorship practices that violate established authorship criteria. The problems discussed here are not new—concerns about authorship integrity have been documented for decades. What has changed is the *scale and systematization* of these practices, driven by increasingly dysfunctional institutional incentive structures. By synthesizing empirical evidence on authorship trends, documented prevalence of misconduct, and systemic drivers, evidence presented in this synthesis demonstrates that contemporary academic culture has created conditions in which authorship inflation thrives at the expense of accountability, equity, and trust in science.

## Methods and approach to synthesis

This commentary synthesizes evidence from peer-reviewed literature on authorship practices, publication pressure, and research integrity. Literature was systematically identified through searches in PubMed, Scopus, and Web of Science databases using key terms: 'authorship,' 'honorary authorship,' 'gift authorship,' 'publication pressure,' 'research integrity,' 'authorship misconduct,' and related terms. Inclusion criteria were: (1) empirical studies or systematic reviews; (2) primary focus on authorship practices, publication pressure, or research misconduct; (3) publication in peer-reviewed journals; (4) studies primarily from 2000-2025, with foundational work from earlier decades included. Data from included studies were synthesized thematically to identify common findings regarding authorship trends, prevalence of misconduct, and documented systemic drivers.

This approach provides systematic, evidence-based synthesis of existing peer-reviewed literature rather than opinion-based commentary on the topic.

## The empirical evidence: quantifying authorship inflation

### The decline of single-author publications

Multiple large-scale bibliometric analyses document the systematic decline of single-author publications across disciplines and geographic regions. Global analyses of publication patterns reveal significant reductions in solo-authored work, with particularly dramatic declines in fields emphasizing collaborative research [9]. However, disciplinary context substantially modulates these trends. Some studies document that humanities disciplines maintain substantially higher proportions of single-authored publications (remaining above 60% in some fields), while social sciences show more balanced distributions of single and collaborative authorship [2, 4]. By contrast, natural sciences, medicine, and engineering have experienced near-complete shifts to multi-authorship, with average article authorships exceeding 5–10 authors. These disciplinary variations reflect fundamental differences in research methodology, funding structures, and publication traditions (e.g., monographs remain predominant in humanities while journal articles dominate in sciences) rather than uniform responses to institutional pressures. Yet across all disciplines, the shift toward increased co-authorship persists, reflecting broader structural forces operating across the global scientific enterprise.

Recent documentation reveals acceleration of these trends. More than 9,000 researchers worldwide now publish at least 72 papers in a single year, highlighting a dramatic rise in hyperprolific authorship in academia [18]. While some hyperprolific output reflects legitimate large-scale collaborations, survey studies document that 18–57% of authors do not meet International Committee of Medical Journal Editors (ICMJE) criteria [25, 33], raising questions about whether this output reflects proportional intellectual contribution. Or has the pressure to publish transformed authorship attribution into a strategic resource to be distributed, traded, and accumulated?

### Growth in authorship counts and the emergence of organized misconduct

The increase in author numbers per publication extends beyond collaborative research advances to include deeply concerning patterns. Paper mills—organized commercial enterprises selling authorship slots on scientific papers—represent a quantifiable but variably documented threat to research integrity. Prevalence estimates vary substantially by discipline and detection methodology, with machine-learning based textual analysis suggesting that suspected paper-mill-associated publications constitute 3–15% of biomedical literature depending on field specificity and detection stringency [37], though only approximately 0.15% of systematic reviews published 2013–2024 directly cite retracted paper-mill articles [40]. These operations submit manufactured manuscripts, often co-opting researchers for financial gain or to inflate publication and citation metrics, resulting in systematic addition of non-contributing authors to publications regardless of intellectual input [22, 37]. Paper-mill networks differ structurally from legitimate collaborative networks: researchers associated with paper-mill outputs tend to have co-authorship networks with unusually low clustering coefficients and geographically concentrated contributions, reflecting the transactional rather than intellectual nature of their collaborations [32, 37].

The existence of citation cartels—groups of authors or journals that systematically cite each other's work to artificially inflate perceived impact and credibility—further compounds these problems [11, 46]. Such cartels frequently overlap with fabricated authorship networks and undermine the reliability of citation-based research metrics. The implications for research assessment and trust in science are profound: inflated authorship and citation patterns distort the evaluation of scientific output, elevate undeserving participants, and hinder identification of legitimate contributions. Yet despite calls for targeted interventions—including algorithmic detection of abnormal network properties and enhanced transparency in authorship attribution [22, 32]—these practices persist and, by most accounts, are growing.

## The problematic dimensions: honorary, gift, and coercive authorship

### Prevalence of honorary and gift authorship: a persistent problem

Honorary authorship—where individuals receive authorship credit without making substantial contributions meeting established criteria—remains one of the most common forms of publication misconduct. The empirical evidence is sobering. Surveys indicate that approximately one in four researchers in the United States and Canada have included honorary authors on their publications [33]. When stringent ICMJE criteria—which hold formal jurisdiction primarily within medical publishing—are rigorously applied, the proportions become even more alarming: perceived honorary authorship prevalence of 18.0% increases to 55.2% when strictly using ICMJE-defined criteria in postgraduate medical research [33]. Recent systematic reviews across health sciences report pooled prevalence rates of 18% for perceived honorary authorship (with ICMJE criteria explained to respondents) and 51% for ICMJE-defined honorary authorship [25]. Beyond health sciences, alternative authorship frameworks operate in humanities, social sciences, and engineering disciplines, though documented prevalence of honorary authorship appears substantial across scholarly fields regardless of formal guideline adoption. Comprehensive reviews of research on authorship meaning, ethics, and practices across scholarly disciplines reveal that many researchers view reciprocal authorship exchanges as acceptable professional courtesies rather than ethical violations, suggesting normalization of practices that violate established authorship criteria [23].

Longitudinal documentation reveals that authorship misconduct concerns have been systematically documented in peer-reviewed literature for over three decades [3, 34, 38]. Rather than declining, empirical evidence indicates that these practices have persisted and in many cases intensified despite widespread availability of guidelines, ethical frameworks, and research integrity initiatives.

### Publication cartels and coercive authorship: interconnected mechanisms

Strategic co-authorship manifests through interconnected mechanisms that exploit both network reciprocity and hierarchical power imbalances. Publication cartels—organized groups systematically adding each other to publications regardless of contribution—have been analyzed through simulation frameworks. Barta [5] developed an agent-based simulation revealing that cartel members can significantly boost productivity even when institutional metrics employ fractional weighting (1/n rule), particularly when external collaboration exists. These self-reinforcing networks form through reciprocal authorship exchanges, where scholars mutually invite each other as co-authors regardless of actual involvement, with powerful incentives for sustained participation. Both productive and minimally-contributing cartel members benefit equally from metric accumulation, making these arrangements difficult to disrupt through metric reforms alone [5, 45]

Coercive authorship operates through mechanisms distinct from but intertwined with publication cartels. While cartels rely on consensual reciprocal exchange among relatively equal-status researchers (colleagues mutually inviting each other), coercive authorship exploits power asymmetries: 28–34% of students across disciplines report compelled inclusion of senior colleagues or supervisors lacking qualifying contributions [14]. Critically, these mechanisms reinforce one another: cartels provide institutional legitimacy for expanded authorship networks (making collaborative inclusion normative), while coercive hierarchies ensure that junior researchers comply with such networks despite recognizing their ethical problems. A senior researcher can simultaneously participate in cartel networks (reciprocal exchange with peers) while coercing junior collaborators into non-contributing authorship. This layering creates compounding disadvantages for junior researchers, who must navigate both explicit power demands and implicit pressure to participate in broader authorship-exchange ecosystems [14, 45].

The prevalence of coercive authorship varies significantly by discipline and geography, ranging from 10% in law to 49% in medical sciences, and from 19% in Hungary to 43% in Portugal [14]. The most common justification students provided was stark in its simplicity: "the person in power requested inclusion as co-author." This highlights how hierarchical structures enable exploitation, even when the exploited recognize the ethical problems inherent in such demands. Junior investigators face impossible choices: resist and risk jeopardizing careers, access to resources, and future opportunities, or comply and perpetuate a system they know to be unethical. Research documents that these overlapping pressures compound knowledge gaps about authorship criteria, creating structural vulnerability that individual ethics training cannot resolve [3].

## Systemic drivers: the "publish or perish" imperative

### Publication pressure and research misconduct: creating the conditions for compromise

Multiple empirical investigations document that strategic co-authorship proliferation correlates with specific institutional and systemic features. Surveys reveal that 71% of researchers observe publication pressure within their institutions, with honorary authorship identified as the most common form of research misconduct [29]. Critically, perceived publication pressure correlates significantly with fabrication, falsification, questionable research practices, and, most ominously, researchers' willingness to engage in future scientific misconduct [15, 29, 43]. This suggests that institutional pressures do not merely create opportunities for ethical violations but actively foster their rationalization and normalization.

It is crucial to emphasize that authorship misconduct emerges as a significant problem primarily in contexts of high-volume institutional evaluation and research assessment. At the level of individual publications evaluated by field experts, such misconduct can often be detected: researchers working in specialized communities typically possess sufficient knowledge of major contributors to identify aberrant authorship patterns and evaluate contributions against claimed expertise. The problem becomes acute, however, when institutional assessments must evaluate thousands of publications across dozens of fields without benefit of such field-specific proximity and expertise. As assessment scales aggregate, from single papers to departmental outputs to institutional rankings, the evaluators' "closeness" to the field diminishes, evaluation timelines compress, and opportunities for detailed scrutiny decline. This aggregation creates vulnerability to gaming behaviors: what might be flagged as suspicious in peer-review context becomes invisible when masked within volumetric institutional metrics. It is in these higher-order evaluative scales, not in individual publication quality, that authorship misconduct flourishes. This distinction is critical: the problem is not that individual researchers lack capacity to judge publication quality, but rather that institutional systems have adopted metrics that prevent such judgment from occurring at scale.

Yet despite this extensive documentation, the fundamental structures driving publication pressure (metric-based evaluation, institutional rankings, competitive funding allocation) remain largely intact and, in many contexts, continue to intensify.

### Metric-based evaluation systems: perverse incentives and strategic responses

**Fig. 1:**
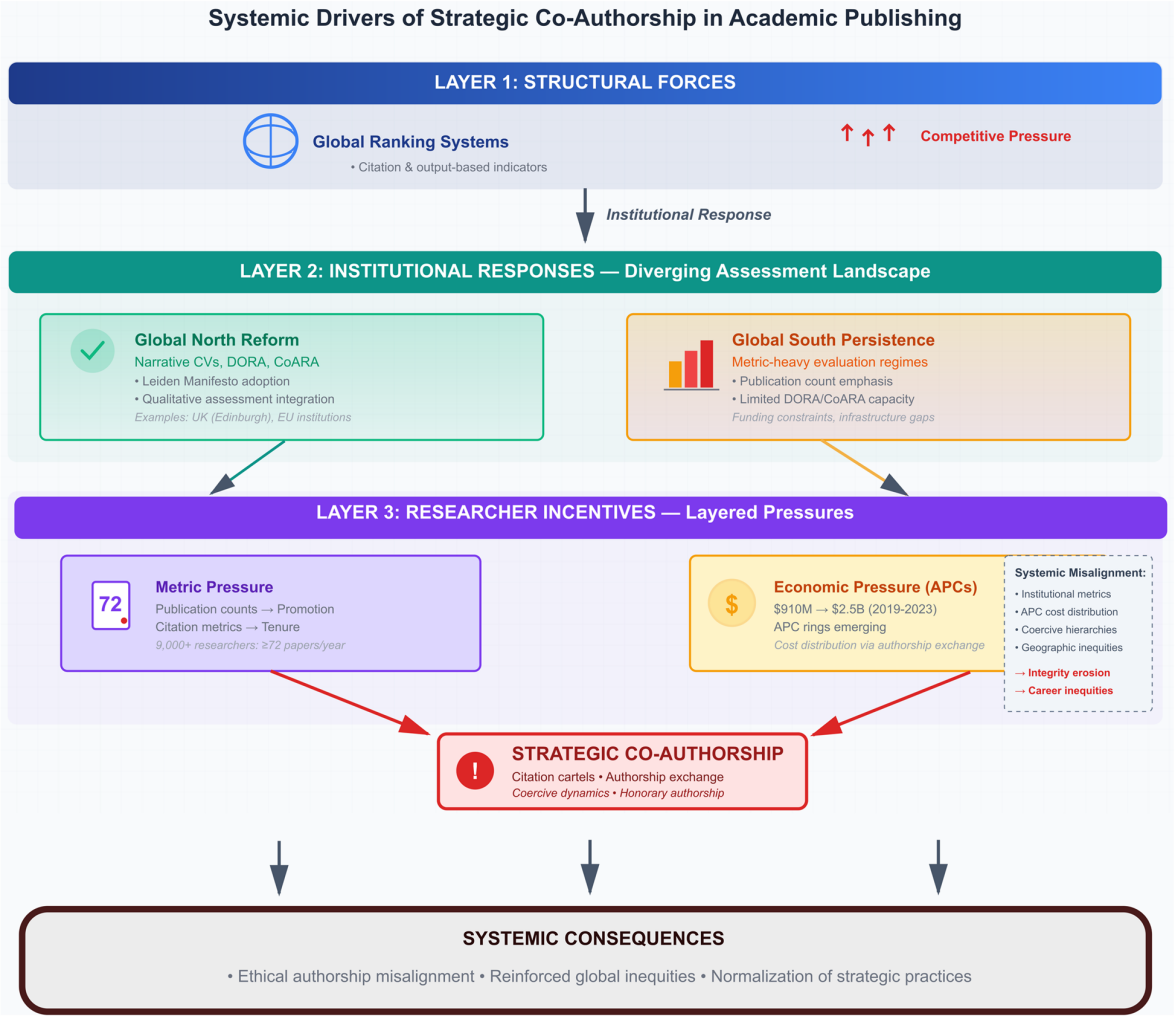
Three-layer systemic model of incentives for strategic co-authorship in academic publishing. The top layer depicts structural forces, where global ranking systems relying on citation- and output-based indicators create competitive pressure on institutions. The middle layer shows diverging institutional responses, with reforms toward narrative CVs and qualitative assessment in parts of the Global North contrasted with persistent, metric-heavy evaluation regimes in many Global South contexts. The bottom layer illustrates how metric pressure (publication and citation counts) and economic pressure (APCs and “APC rings”) jointly incentivize strategic co-authorship practices (such as citation cartels, authorship exchange, and coercive or honorary authorship) culminating in systemic consequences for ethical authorship alignment, global equity, and the normalization of these practices

Research institutions employ publication metrics for personnel evaluation through distinct pathways. Large-scale analyses of review, promotion and tenure policies indicate that quantitative, metric-based assessment (for example, publication counts, journal metrics and citation indicators) is now widespread across institutions in multiple countries [20, 31]. However, it is important to distinguish institutional choice from structural compulsion: universities often integrate such metrics in response to competitive pressures and global ranking systems that privilege citation-based and publication-count indicators, rather than as purely autonomous design choices [20, 31]. Universities that resist such metrics face competitive disadvantage in global rankings, creating structural pressure to adopt these assessment methods regardless of leadership preferences [39]. This creates a reflexive cycle: global rankings drive institutional adoption of metrics, institutional adoption of metrics increases researcher pressure to maximize output, and researcher incentive structures systematically favor strategic co-authorship (see Fig. 1).

Analysis of 143 tenure and promotion policies across seven countries reveals a diverging landscape: while higher-income institutions in the Global North are progressively incorporating narrative CVs and qualitative assessment alongside quantitative measures, bibliometric-heavy evaluation remains predominant in Global South institutions [31]. This is particularly acute in contexts where research assessment reform initiatives like DORA (Declaration on Research Assessment) or CoARA (Coalition for Advancing Research Assessment) have limited implementation capacity, perpetuating metric-driven incentive structures that directly incentivize the strategic co-authorship practices discussed here. Recent global assessment reform initiatives (DORA, CoARA, the Leiden Manifesto) have gained traction primarily in the Global North. UK universities (e.g., University of Edinburgh) and Continental European institutions now pilot narrative CVs and explicitly abandon Journal Impact Factor in recruitment and tenure decisions [30]. Conversely, this reform momentum has not uniformly reached Global South institutions, where funding constraints and infrastructure limitations perpetuate bibliometric-heavy assessment regimes. Qualitative studies document that researchers in the Global South experience strong publication pressures tied to institutional expectations for publication in high-impact Global North journals, a structural inequity that reinforces Global North epistemic and evaluative dominance [28]. The result is that strategic co-authorship remains systemically incentivized in precisely those contexts where reform capacity is lowest, exacerbating existing research equity gaps.

Investigations of citation metrics and authorship practices demonstrate that current assessment systems create both opportunities and incentives for strategic manipulation [10]. Network analyses of citation patterns reveal researchers engaging in coordinated citation practices and reciprocal authorship arrangements designed to boost metrics [10]. These "citation cartels" operate similarly to authorship cartels, exploiting evaluation systems that treat citations and authorship as objective quality indicators while failing to account for strategic manipulation. This suggests that reforming counting methods alone cannot address strategic authorship without broader changes to assessment philosophies and institutional cultures.

This represents a structural misalignment: current assessment systems create incentives for strategic authorship output, while researchers adhering to ICMJE criteria, or other equivalente authorship framework in other disciplines, face competitive disadvantages within the same evaluation framework. Empirical evidence suggests that metric-based incentive structures, absent fundamental reform, generate pressures that rationalize strategic co-authorship practices, with institutional promotion and recognition reinforcing rather than deterring such behavior.

### Open access economics and authorship incentives

The transition toward Open Access publishing introduces additional incentive mechanisms beyond institutional metrics. Article Processing Charges (APCs)—fees ranging from €500 to €5,000 + per article—have become significant cost items within research budgets. With global APC spending nearly tripling from $910.3 million (2019) to $2.538 billion (2023), researchers with access to grant-funded OA budgets face intensified pressures to maximize publication output [16]. In 2023, MDPI, Elsevier, and Springer Nature generated the largest APC revenues ($681.6 M, $582.8 M, and $546.6 M respectively), concentrating costs among well-resourced institutions [16].

This economic structure creates novel authorship incentives. Research teams with APC funding face pressure to maximize publication output to justify costs, analogous to traditional metric-driven practices. Moreover, emerging networks termed "APC rings" operate through strategic authorship exchange to distribute publication costs across multiple authors and institutions [21, 41]. While distinct from traditional publication cartels, these structures exploit similar mechanisms: authorship as a currency to be exchanged rather than as meaningful attribution of contribution.

The result is a layered incentive system: researchers pursue high publication counts for institutional advancement while simultaneously managing APC costs through collaborative networks. For junior researchers and those from lower-income institutions, this dual pressure intensifies the coercive authorship dynamics already documented in this synthesis.

## Current mitigation efforts and their limitations: why existing solutions fall short

### Guidelines and taxonomies: transparency without enforcement

Current mitigation strategies (ICMJE criteria or CRediT taxonomy) offer structured frameworks but fail as meaningful interventions against strategic authorship due to absent verification and enforcement. The ICMJE defines authorship through four mandatory conditions (substantial contributions to conception/design; drafting/revision; final approval; full accountability), yet implementation varies widely across journals, with most relying on unverified author attestation rather than independent validation [8, 24]. Authorship disputes remain resolved by senior authors or conflicted institutions lacking procedural independence [35].

CRediT taxonomy, adopted by major publishers (Elsevier, Springer Nature, Wiley), standardizes 14 contribution roles but explicitly does *not* define authorship thresholds, serving descriptive rather than preventive functions [6]. While providing transparency for honest attribution, CRediT assumes good faith, offers no verification mechanisms, and cannot deter misrepresentation when power dynamics or strategic incentives prevail [1]. Both systems share fundamental limitations: ICMJE lacks jurisdiction beyond medicine and enforcement will; CRediT provides "stop-and-think" utility at best but no structural barriers to unethical practices.

## Consequences for research integrity and scientific progress: what we stand to lose

### Accountability and attribution challenges: who is responsible?

When authorship does not accurately reflect contribution, accountability for research quality and integrity becomes diffused and difficult to assign. Analyses of the relationship between authorship practices and research misconduct investigations reveal that honorary and gift authorship complicate efforts to identify responsible parties when problems emerge [19]. All authors ostensibly share responsibility for published work, yet honorary authors may have no familiarity with methods, data, or analyses, creating accountability gaps that undermine research integrity infrastructure.

These concerns are not new. Already in 1997, fundamental examinations of authorship attribution documented how unclear authorship responsibilities affect the integrity of scientific publishing and create challenges for accountability [38]. The persistence of this problem, documented for nearly three decades without resolution, suggests that authorship inflation creates not only ethical issues at publication but also enduring practical challenges for maintaining the integrity of the scientific record over time. Longitudinal data spanning three decades document persistent authorship misconduct despite intervention efforts. The absence of documented improvement and evidence of intensification in some contexts, suggests that current approaches have not achieved their intended effects.

### Inequities and career implications: systematic disadvantage

Honorary and gift authorship create systematic inequities in competition for publications, which translate into unfair advantages in job applications, promotion decisions, and salary determinations. This practice provides unearned advantages to those included while disadvantaging researchers who adhere to ethical standards. The impacts fall disproportionately on early-career researchers, who compete against colleagues benefiting from strategic authorship arrangements. Documentation of authorship challenges facing junior investigators reveals how pressure to participate in unethical practices versus career disadvantages from refusing creates impossible dilemmas [12, 19]. This suggests that authorship misconduct is not merely an individual ethical issue but a structural problem that *systematically disadvantages* those committed to integrity, effectively penalizing ethical behavior while rewarding misconduct.

### Erosion of trust in science: the broader implications

Perhaps most fundamentally, widespread authorship misconduct undermines public and scientific community trust in the research enterprise. Investigations of how research integrity violations affect trust reveal that awareness of misconduct (including authorship violations) significantly reduces confidence in scientific findings among both researchers and the public [42]. When authorship attribution becomes unreliable, it becomes difficult to assess researchers' genuine expertise, evaluate their qualifications for positions or grants, or determine appropriate reviewers for manuscripts.

The recent increase in paper mill operations and revelations of fabricated authorship networks have heightened public skepticism [7]. While paper mills represent an extreme form of misconduct distinct from the strategic co-authorship practices discussed here, they highlight vulnerabilities in current systems and contribute to broader erosion of trust that affects all scientific publishing. In an era when science faces unprecedented challenges from misinformation, politicization, and public skepticism, public and scientific community trust in research is documented to decline when awareness of research misconduct (including authorship violations) increases [42]. Persistent authorship misconduct may therefore compromise the research enterprise's credibility and public trust at a time when both are increasingly vulnerable.

## Recommendations: a multi-level reform agenda

### Institutional assessment reform: changing the incentives

Universities and research organizations must fundamentally reconsider evaluation and reward systems. The overwhelming emphasis on publication quantity and citation metrics creates conditions in which strategic co-authorship thrives. The Leiden Manifesto articulates principles for responsible research metrics, emphasizing the need for qualitative assessment alongside quantitative indicators, recognition of diverse contributions beyond traditional publications, and evaluation practices that respect disciplinary differences [17]. Implementation of these principles would reduce pressures driving unethical authorship practices, yet adoption remains frustratingly slow despite widespread endorsement.

Specific reform proposals include limiting the number of publications considered in evaluation dossiers, requiring detailed contribution statements for all listed works, and shifting emphasis toward real-world impact rather than proxy metrics [26]. Survey evidence confirms widespread support (91% of researchers endorse assessment reforms [36]), suggesting barriers are institutional and political rather than technical.

### Editorial and publisher interventions: raising the barriers

Scientific journals occupy critical positions in the publication ecosystem and can implement important measures to detect and deter authorship misconduct. Major commercial and society publishers now maintain detailed authorship and ethics policies, provide editors with procedural guidance on handling authorship disputes and misconduct, and in many cases actively enforce these standards through investigative workflows and corrective actions. Proposals that journals require structured contributorship statements, individual confirmation of contributions from all authors, not simply attestation by the corresponding author, and verification procedures during manuscript handling would enhance transparency and accountability [34]. Although resource-intensive, these practices would raise meaningful barriers to honorary, gift, and strategic authorship.

Editors should receive training to recognize patterns characteristic of authorship misconduct, including unusually high author counts relative to study type, sudden appearance of new collaborator networks, and citation patterns suggesting coordinated manipulation [27]. Documentation confirms that editorial vigilance can effectively identify problematic authorship through pattern recognition and targeted inquiry [27]. Systematic application of such approaches could deter misconduct by increasing detection likelihood. However, sustaining this level of editorial oversight requires ongoing investment of time and resources, and many journals must balance strengthened authorship policing against financial and workload constraints, so that implementation remains uneven across the ecosystem.

### Cultural and mentorship change: modeling integrity

Ultimately, addressing authorship inflation requires fundamental cultural change in how the academic community values and rewards research contributions. Research on mentorship in transmitting research integrity norms documents that early-career researchers take cues about acceptable behavior from supervisors and senior colleagues [3]. When senior researchers model ethical authorship practices and protect junior colleagues from coercive pressures rather than exploiting power imbalances, they create environments supporting integrity. However, when senior researchers participate in or enable strategic authorship, they signal to junior colleagues that such practices are acceptable professional behavior.

Transparent discussion of authorship expectations should occur at the inception of research projects, with written agreements documenting anticipated contributions and authorship order. Systematic reviews demonstrate that clear authorship policies and early agreements can reduce disputes and promote honest attribution [23]. Making such agreements standard practice would create accountability mechanisms that deter strategic inclusion of non-contributing authors. Yet the effectiveness of such agreements depends on power dynamics: junior researchers may feel unable to resist unreasonable authorship demands even when formal agreements exist.

## Conclusion: the path forward

The near-disappearance of single-author publications in scientific literature represents more than a neutral evolution toward collaborative science. While genuine collaboration should be celebrated and facilitated, substantial evidence demonstrates that publication pressures and metric-based evaluation systems have incentivized strategic co-authorship practices that violate established criteria and undermine research integrity. The scale of the problem is sobering: honorary authorship affects approximately one-third to one-half of publications in some fields (depending on how strictly criteria are applied), coercive authorship impacts nearly one-third of doctoral students globally, and publication cartels have emerged as organized responses to dysfunctional assessment systems. Despite decades of intervention efforts [3, 34, 38], prevalence data indicate persistence or intensification, suggesting fundamental inadequacy of existing strategies.

These findings suggest multiple leverage points for institutional response. Research assessment reform—limiting publications counted in evaluation, requiring detailed contribution statements, and shifting emphasis toward qualitative impact—could reduce publication-pressure incentives [17]. Enhanced editorial verification procedures, training of editors to recognize authorship patterns characteristic of misconduct, and mandatory contributor role attribution could raise barriers to honorary and coercive authorship [27, 34]. Cultural shifts in how the academic community values mentorship and rewards integrity (rather than exploiting power asymmetries) could reshape norms around authorship inclusion [3, 23]. Empirical evidence from institutions implementing such reforms (e.g., DORA signatories, CoARA participants) indicates these approaches can reduce strategic authorship incentives, though implementation capacity varies substantially by region and institutional resource availability [31].

Most fundamentally, the academic community must acknowledge that current incentive structures systematically undermine the principle that authorship should reflect genuine intellectual contribution. The integrity of science depends on honest attribution of scholarly work. When publication counts matter more than contribution quality, when metrics can be gamed through strategic arrangements, and when power imbalances enable exploitation, the scientific enterprise loses its foundational commitment to truth and accountability.

The evidence presented in this synthesis demonstrates that current institutional incentive structures—metric-based evaluation, competitive funding allocation, and hierarchical power asymmetries—create conditions under which authorship inflation persists despite documented availability of alternatives. The integrity of the scientific record depends on accurate attribution of intellectual contribution; when publication quantity supersedes quality assessment, when metrics remain susceptible to strategic manipulation, and when power imbalances limit junior researchers' agency, accountability becomes diffused and misconduct becomes rationalized.

Survey evidence documents widespread endorsement of research assessment reform and existence of documented institutional models that reduce metric-driven incentives. The barriers to broader implementation appear to be institutional and political rather than technical or conceptual. Whether this implementation gap can be narrowed depends on whether institutional decision-makers assign priority to research integrity over competitive positioning in global ranking systems: a structural choice rather than a knowledge gap. The empirical record demonstrates that strategic co-authorship will likely persist and intensify absent systematic change to the incentive structures that currently sustain it.

## Data Availability

All data supporting the findings of this commentary are derived from published peer-reviewed literature, as referenced throughout the manuscript. No new datasets were generated or analyzed for this study.
